# Rare causes of genital fistula in nine African countries: a retrospective review

**DOI:** 10.1186/s12905-022-02050-z

**Published:** 2022-12-06

**Authors:** Carrie J. Ngongo, Thomas J.I.P. Raassen, Marietta Mahendeka, Ladeisha Lombard, Jos van Roosmalen, Marleen Temmerman

**Affiliations:** 1 Global Health Division, RTI International, Research Triangle Park, USA; 2 Nairobi, Kenya; 3 Bugando Medical Centre, Mwanza, Tanzania; 4 Cape Town, South Africa; 5 Leiden University Medical Centre and Athena Institute VU University, Amsterdam, the Netherlands; 6 Centre of Excellence in Women and Child Health, Aga Khan University, Nairobi, Kenya; 7 Faculty of Medicine and Health Sciences, Ghent University, Ghent, Belgium

**Keywords:** Fistula, Injury, Traditional healer, Trauma

## Abstract

**Background:**

Most genital fistulas result from prolonged, obstructed labor or surgical complications. Other causes include trauma (from accidents, traditional healers, or sexual violence), radiation, carcinoma, infection, unsafe abortion, and congenital malformation.

**Methods:**

This retrospective records review focuses on rare fistula causes among 6,787 women who developed fistula after 1980 and sought treatment between 1994 and 2017 in Tanzania, Uganda, Kenya, Malawi, Zambia, Rwanda, Ethiopia, Somalia, and South Sudan. We compare fistula etiologies across countries and assess associations between rare causes and type of incontinence (urine, feces, or both).

**Results:**

Rare fistula accounted for 1.12% (76/6,787) of all fistulas, including traumatic accidents (19/6,787, 0.28%), traumatic sexual violence (15/6,787, 0.22%), traumatic injuries caused by traditional healers (13/6,787, 0.19%), unsafe abortion (10/6,791, 0.15%), radiation (8/6,787, 0.12%), complications of HIV infection (6/6,787, 0.09%), and congenital abnormality (5/6,787, 0.07%). Trauma caused by traditional healers was a particular problem among Somali women.

**Conclusion:**

Fistulas attributable to rare causes illuminate a variety of risks confronting women. Fistula repair training materials should distinguish trauma caused by traditional healers as a distinct fistula etiology. Diverse causes of fistula call for multi-pronged strategies to reduce fistula incidence.

**Supplementary Information:**

The online version contains supplementary material available at 10.1186/s12905-022-02050-z.

## Background

Most genital fistulas are obstetric, arising from pressure necrosis during prolonged, obstructed labor. Obstetric fistula poses a challenge in remote, low-resourced settings. Obstetric fistula has been nearly eliminated everywhere where women have sufficient and timely access to quality emergency obstetric care.

While most genital fistulas follow childbirth in low-resourced settings, fistulas in well-resourced settings occur primarily as surgical complications [1, 2]. A rising proportion of genital fistulas may be attributable to surgical accidents in low-resource settings as well [3–5].

Other genital fistula etiologies have been reported. FIGO’s 2011 Fistula Surgery Training Manual cited traumatic causes (including [forceful] coitus, sexual violence, accidental trauma, and female genital mutilation), infection (including granulomatous tuberculosis, and HIV), malignancy (especially advanced cervical cancer), radiotherapy, and congenital malformations [6].

We examined the circumstances leading to fistula development in a separate analysis, focusing on genital fistulas following vaginal birth, cesarean birth, and gynecological surgery in nine African countries [7]. In this article we seek to document rare causes of genital fistula. Fistulas attributable to rare causes illuminate a variety of risks confronting women. Clarity about these causes and risks can help policymakers to develop appropriate strategies to reduce fistula incidence.

## Methods

This retrospective records review evaluated the causes of genital fistulas in 6,787 women who sought fistula treatment in nine African countries, focusing on rare causes other than childbirth and surgical complications. The second and third author and colleagues interviewed and operated women in 89 facilities in Tanzania, Uganda, Kenya, Malawi, Rwanda, Somalia, South Sudan, Zambia, and Ethiopia. One of the surgeons interviewed each woman and recorded information on a standard form [4]. Women consented to fistula repair surgery in their respective hospitals, following each hospital’s counseling and informed consent process. We operated in multiple facilities offering fistula repair to wide catchment areas (Table S1). The number of women in the different countries reflect the number of repairs conducted by the second and third author and colleagues in those countries. Data were collected between June 1994 and December 2017.

We excluded 16 women whose fistulas were of unclear origin. We excluded 248 women whose fistulas developed before 1980, given small sample sizes of fistulas that developed 15 or more years before the earliest date of data collection, which was at the time of fistula treatment. These 248 excluded records included only one woman with fistula that developed before 1980 from a cause other than childbirth or surgery. We excluded six women with “postrepair” fistulas that resulted from fistula surgery.

We grouped genital fistulas according to the circumstances of fistula development. Fistulas following childbirth or surgery were separately investigated [7]. We divided traumatic fistulas between those related to sexual violence, traditional healers, and accidental trauma. Inserted foreign bodies were counted in one of the traumatic categories, depending on context. Other rare fistula causes included abortion, radiation, complications of HIV infection, and congenital abnormality. We compared frequencies in these categories of causes across the nine countries. We also provided associations between incontinence type (vesico-vaginal fistula, recto-vaginal fistula or both) and rare fistula causes.

Data were entered into an Excel database, with names changed to unique identification numbers to protect privacy. Data were analyzed using Stata 16 software (StataCorp). Approval for this record review was granted by the AMREF Ethics and Scientific Review Committee (P88/2013).

## Results

This analysis focuses on 76 (1.12%) women with fistula causes not attributable to childbirth or surgery among 6,787 women seeking fistula repair in nine countries. This 1.12% of the fistula burden was attributable to traumatic accidents (19/6,787, 0.28%), traumatic sexual violence (15/6,787, 0.22%), traumatic injuries caused by traditional healers (13/6,787, 0.19%), unsafe abortion (10/6,787, 0.15%), radiation (8/6,787, 0.12%), complications of HIV infection (6/6,787, 0.09%), and congenital abnormality (5/6,787, 0.07%) (Table 1).

**Table 1:** Rare fistula causes by country

**Fistula Cause**	**Tanzania**		**Uganda**		**Kenya**		**Malawi**		**Zambia**	
Traumatic accident	4	0.18%	5	0.33%	4	0.35%	3	0.45%	-	-
Traumatic sexual violence	3	0.14%	4	0.26%	4	0.35%	-	-	-	-
Traumatic traditional healer	3	0.14%	-	-	1	0.09%	-	-	-	-
Unsafe abortion/Dilatation and curettage	3	0.14%	-	-	4	0.35%	1	0.15%	-	-
Radiation	4	0.18%	-	-	2	0.18%	1	0.15%	1	0.60%
Complication of HIV infection	1	0.05%	1	0.07%	2	0.18%	2	0.30%	-	-
Congenital	1	0.05%	1	0.07%	1	0.09%	1	0.15%	1	0.60%
**Women with fistula from rare causes**	**19**	**0.86%**	**11**	**0.72%**	**18**	**1.59%**	**8**	**1.21%**	**2**	**1.19%**
**Women with fistula**	**2,213**		**1,527**		**1,131**		**662**		**168**	
**Fistula Cause**	**Rwanda**		**Ethiopia**		**Somalia**		**South Sudan**		**Total**	
Traumatic accident	1	0.24%	2	1.82%	-	-	-	-	19	0.28%
Traumatic sexual violence	-	-	2	1.82%	1	0.32%	1	0.41%	15	0.22%
Traumatic traditional healer	-	-	1	0.91%	8	2.53%	-	-	13	0.19%
Unsafe abortion/Dilatation and curettage	1	0.24%	-	-	-	-	1	0.41%	10	0.15%
Radiation	-	-	-	-	-	-	-	-	8	0.12%
Complication of HIV infection	-	-	-	-	-	-	-	-	6	0.09%
Congenital	-	-	-	-	-	-	-	-	5	0.07%
**Women with fistula from rare causes **	**2**	**0.48%**	**5**	**4.55%**	**9**	**2.85%**	**2**	**0.83%**	**76**	**1.12%**
**Women with fistula**	**418 **		**110 **		**316 **		**242 **		**6,787 **	

Traumatic accidents included falls, traffic accidents, gunshot wounds, and other injuries. Sexual violence included two cases of rape with multiple perpetrators, two cases involving the insertion of a foreign body, and one case targeting a prepubescent girl.

Trauma caused by traditional healers was a particular problem among Somali women. Eight Somali women had burns caused by traditional healers inserting hot iron rods in the vagina for complaints including itchiness, pelvic pain, painful genitalia, “vaginal growth,” “hemorrhoids,” swelling, and infertility. These women accounted for 2.53% (8/316) of genital fistulas repaired in Somalia. Injuries from traditional healers did not account for more than 0.15% of fistulas repaired anywhere else (Table 1).

Unsafe abortion or dilatation and curettage led to fistula development in 10 women, of whom four were in Kenya and three in Tanzania. Radiation-associated fistulas were seen in Tanzania (4), Kenya (2), Malawi (1), and Zambia (1). Fistulas arising as complications of HIV infection occurred in Kenya (2), Malawi (2), Uganda (1), and Tanzania (1). Of these, two developed in infant girls born to HIV-positive mothers. The other four cases were adult women; one had comorbid tuberculosis that may have contributed to fistula formation. Five girls and women had congenital fistula.

Trauma from sexual intercourse most commonly created recto-vaginal fistula (9), although four women developed vesico-vaginal fistula and two developed both vesico-vaginal and recto-vaginal fistula after forceful sexual intercourse. Most radiation-associated fistulas were vesico-vaginal, as were all congenital fistulas. All fistulas arising as a complication of HIV infection were recto-vaginal (Table 2).

**Table 2:** Associations between location and rare fistula causes

Fistula following	Vesico-vaginal fistula and recto-vaginal fistula		Vesico-vaginal fistula		Recto-vaginal fistula		Total
Traumatic accident	-		17	89%	2	11%	19
Traumatic sexual violence	2	13%	4	27%	9	60%	15
Traumatic traditional healer	-		9	69%	4	31%	13
Unsafe abortion/Dilation and curettage	1	10%	8	80%	1	10%	10
Radiation	1	13%	7	88%	-		8
Complication of HIV infection	-		-		6	100%	6
Congenital	-		5	100%	-		5
**Total**	**4**	5%	**50**	66%	**22**	29%	**76**

## Discussion

Many case series have insufficient sample sizes to draw conclusions about rare fistula causes. This relatively large dataset offers a unique opportunity to assess the etiology of fistula beyond the dominant causes of childbirth and obstetric and gynecological surgery. Fistulas attributable to rare causes illuminate a variety of risks confronting women.

In this series the most common rare causes were traumatic, whether attributable to accidents, sexual violence, or traditional healers. Among these, traumatic fistulas from accidents were most common, especially due to falls and road traffic accidents.

Some rare causes involve a person directly doing something harmful to women’s bodies, including unsafe abortion, sexual violence, and treatments from traditional healers. In our experience, fistulas following these circumstances tend to be larger and more difficult to repair than the other rare fistulas. In particular, the average size of fistulas following unsafe abortion is similar to fistulas that develop from pressure necrosis during prolonged, obstructed labor. Following in average size are fistulas attributable to traditional healers, which often involve stenosis and scarring, and fistulas following sexual violence. Fistulas from other causes are typically smaller and thus more similar in size to iatrogenic fistulas from surgical complications. Though generally not large, fistulas from radiation can be difficult to repair because of damage to the surrounding tissue.

Traumatic fistula resulting from sexual violence has been of particular interest in the Democratic Republic of the Congo, where it accounts for 2–3% of fistulas [8–10]. Some hypothesize that female genital cutting predisposes women to fistula given population-level statistical associations [11]. Although it is included as a potential fistula cause in the FIGO fistula training manual, we did not identify any woman with fistula associated with female genital cutting. Ritual female genital cutting such as clitoridectomy or excision does not generally create fistulas [12, 13]. Traditional treatments that cut the anterior vaginal wall can cause fistulas, however. Known as the gichiri or gurya cut, it has been documented in Nigeria and Niger, where it accounts for up to 18% of treated genitourinary fistulas [14–16].

Other fistulas caused by traditional treatments have not received similar recognition, such as the burns from hot iron rods seen in Somalia. Such burns are a specific form of “traditional, damaging, and dangerous [Somali] practices” of fire-burning body parts with coal, iron rods, or burning sticks [17]. Traditional healers may create fistulas when they burn the vagina with hot iron rods to address urinary tract infections or vaginal itchiness [18]. Our review can add other reasons for intentional vaginal burning to this list, including hemorrhoids, swelling, infertility, and various pelvic or genital pains. In Somalia and Kenya, health education and outreach should stress that intentional vaginal burning is not an effective therapy and in fact causes substantial harm.

Traditional therapeutic interventions were not listed among possible fistula causes in the latest fistula surgery training manual [13]. We recommend inclusion in future editions. Otherwise, fistulas caused by traditional healers may be combined with accidental cases that occur in health facilities. Trauma inflicted by traditional healers is clearly distinct from surgical complications, and separate counting will enable appropriately targeted responses.

The FIGO fistula surgery training curriculum groups all iatrogenic fistulas resulting from pelvic surgery. We see value in distinguishing fistulas following gynecological surgery from fistulas attributable to unsafe abortion. Reports have identified cervico-vaginal fistulas as a complication of induced abortion, with implications for women’s subsequent fertility and risk of obstetric complications [19, 20]. This series includes two fecal fistulas through the uterus after dilatation and curettage for incomplete abortions, as well as one case of combined vesico-vaginal and recto-vaginal fistula following unsafe abortion.

HIV infection can lead to fistula formation in rare cases. Acquired rectovaginal fistulas shortly after birth have been documented as an early manifestation of HIV infection in girls, and most acquired recto-vaginal fistulas in children can be attributed to HIV infection [21, 22]. In adults, seropositive women can develop anorectal sepsis, as seen with the four women in this series who had acquired small recto-vaginal fistulas without relation to pregnancy or birth [23].

Radiation therapy for cervical, endometrial, or multifocal cancer can induce fistula and decrease the elasticity of surrounding tissues [2, 24, 25]. Radiotherapy caused 4.6% (4/87) of fistulas reported by one tertiary fistula center in South Africa; the much lower proportion in our population likely reflects comparatively low access to cancer treatment [26].

This large retrospective review has limitations. Sample sizes reflect variation in where the second and third authors and colleagues repaired fistulas. Countries typically included data from multiple hospitals with wide catchment areas. Countries with large sample sizes, however, may be more representative of all women seeking fistula repair than those with smaller samples. This series includes women who sought surgical treatment for their fistulas. It cannot include women who developed fistula but did not reach treatment centers. Overall etiology may be different among all women with fistula if women with particular fistula causes are more likely than others to reach treatment facilities. We cannot rule out the risk of response bias and recall bias given our dependence on women’s accounts of past events.

## Conclusion

Facilities offering fistula surgery should routinely monitor fistula causes, as this will inform appropriate prevention strategies. Although most genital fistulas in low-resource settings follow childbirth, one can expect that the proportions attributable to surgical complications and rare causes will increase with continued progress toward obstetric fistula elimination. Diverse causes of fistula call for multi-pronged strategies to reduce fistula incidence.

## Supplementary Information

Additional file 1: **Table S1.** Included hospitals offering fistula repair by country.

## Data Availability

Data generated and analyzed during the current study are not publicly available during a period of analysis and dissemination but will be available from the corresponding author on reasonable request.
