# Supplementary material for: Rare causes of genital fistula in nine African countries: a retrospective review
Source: BMC Womens Health. 2022 Dec 6;22:497. doi: 10.1186/s12905-022-02050-z (PMC9724406; doi:10.1186/s12905-022-02050-z)
Supplement: Supplementary file 1 — Table S1. Included hospitals offering fistula repair by country. [file 12905_2022_2050_MOESM1_ESM.pdf]

Table S1. Included hospitals offering fistula repair by country

	Included hospitals	Women with fistula
Tanzania	30	2,213
Uganda	11	1,527
Kenya	26	1,131
Malawi	6	662
Zambia	1	168
Rwanda	2	418
Ethiopia	3	110
Somalia	3	316
South Sudan	7	242
Total	89	6,787
